# An antibiotic-free plasmid vector platform for non-viral DNA delivery

**DOI:** 10.1016/j.gendis.2026.102249

**Published:** 2026-05-23

**Authors:** Dan Zhao, Zakary D. Carden, Yifei Wang, Cole S. Hudson, Perry A. Maza, Dana Cyril-Ramirez, Yong Li

**Affiliations:** Section of Epidemiology and Population Sciences, Department of Medicine, Baylor College of Medicine, Houston, TX 77030, USA; Dan L Duncan Comprehensive Cancer Center, Baylor College of Medicine, Houston, TX 77030, USA; Section of Epidemiology and Population Sciences, Department of Medicine, Baylor College of Medicine, Houston, TX 77030, USA; Section of Epidemiology and Population Sciences, Department of Medicine, Baylor College of Medicine, Houston, TX 77030, USA; Dan L Duncan Comprehensive Cancer Center, Baylor College of Medicine, Houston, TX 77030, USA

**Keywords:** pir-116, Antibiotic-free, DNA plasmid, ORF1p, GLP1

Plasmid DNA remains a foundational modality in non-viral gene delivery for research, gene therapy, and cancer immunization.^1^ However, widely used plasmid backbones still depend on antibiotic resistance genes for selection, creating regulatory challenges and biosafety concerns, and limiting the adoption of safer designs. Commercial antibiotic-free systems exist, but their high licensing costs and restricted flexibility impede academic use and iterative optimization. To address these barriers, we created pIR (Fig. 1A), an antibiotic-free plasmid platform that is small, robust, and scalable, using an R6K γ origin and a Cre-mediated excision mechanism controlled within a specialized *pir*^+^bacterial host.

**Figure 1:**
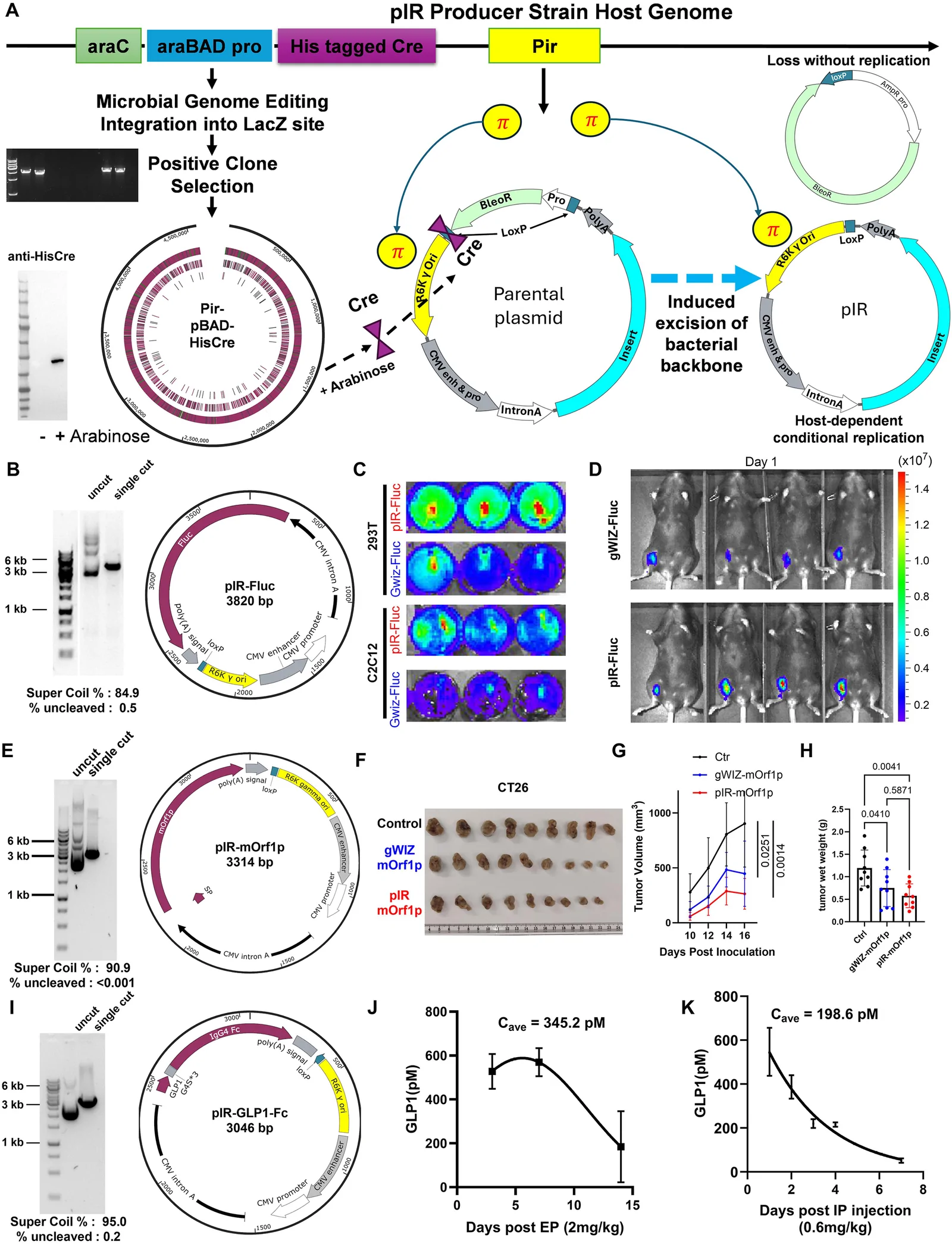
pIR: A new antibiotic-free plasmid production platform for non-viral DNA delivery. **(A)** Illustration of the pIR platform. The system consists of two parts: one is an engineered *E. coli* strain with arabinose-inducible His-tagged Cre recombinase, and the other is a plasmid vector with two loxP sites. Cre is introduced in a pir^+^ strain, which expresses the π protein that is essential for the replication of plasmids containing the γ origin of replication. Successful engineering was confirmed by PCR, whole-genome sequencing, and Western blotting. The antibiotic resistance gene is removed upon arabinose induction, resulting in a vector that can continue to replicate for many generations. **(B)** Enzymatic digestion (left) and the map of pIR-Fluc (right). **(C)** Bioluminescence imaging using the IVIS Lumina X5 system on *in vitro* transfected 293T (top) and C2C12 (bottom) cells with gWIZ-Fluc or pIR-Fluc. **(D)** IVIS images of mice one day post-electroporation with gWIZ-Fluc or pIR-Fluc. gWIZ-Fluc (top) or pIR-Fluc (bottom) was intramuscularly injected and electroporated into 7–9-week-old C57bl/6 mice quadriceps using the Ichor Trigrid device. **(E)** Enzymatic digestion (left) and the map (right) of pIR-mOrf1p. **(F–H)** Tumor image (F), tumor size measurements (G), and tumor wet weights (H) were shown for mice inoculated with 1 million CT26 cells in the right flank and treated with either mock electroporation or electroporation with either gWIZ-mOrf1p or pIR-mOrf1p. **(I)** Enzymatic digestion (left) and map (right) for pIR-GLP1-Fc. **(J, K)** Serum GLP1 concentrations measured by ELISA. Mice were treated with either 2 mg/kg pIR-GLP1-Fc DNA (J) via electroporation at the quadriceps muscle or 0.6 mg/kg dulaglutide peptide (K) via intraperitoneal injection. C_ave_ was calculated by the area under the curve (AUC) divided by the number of days. Two-way ANOVA was used for Figure 1G and one-way ANOVA for Figure 1H. *P*-values were as labeled.

pIR relies on two engineered components. First, a pir-116 strain—selected for its ability to support high-copy R6K γ replication—was modified by integrating an arabinose-inducible Cre recombinase cassette into the *lacZ* locus through CRISPR/Cas9-guided recombination (Fig. S1A–S1C). Whole-genome sequencing confirmed precise insertion without unintended mutations, and Western blotting verified inducible Cre expression (Fig. S1D and S1E). Second, pIR plasmids contain a minimal R6K γ origin and a compact loxP-flanked Bleo^R^ marker. Upon induction, Cre excises the Bleo^R^ cassette, producing a smaller plasmid that is free from an antibiotic-resistant gene yet can persist for long periods without positive selection. For gene expression in mammalian cells, pIR incorporates the full CMV promoter, including intron A and a complete Kozak sequence, maximizing transcription and translation while keeping the overall backbone size extremely small.

Across multiple constructs, pIR preparations consistently yielded high-purity, antibiotic-free DNA with > 99% plasmid cleavage efficiency and maintained 80%–90% supercoiled content (Fig. 1B–E, I; Fig. S2D), an important determinant of delivery efficiency. In reporter assays, pIR demonstrated clear advantages over the widely used gWIZ plasmid. *In vitro*, pIR-Fluc showed roughly 2-fold higher luminescence in 293T cells and 4-fold higher in C2C12 myoblasts (Fig. 1C; Fig. S2A). *In vivo*, intramuscular electroporation of pIR-Fluc (25 μg) produced stronger and more durable bioluminescence than gWIZ-Fluc, persisting for at least 14 days (Fig. 1D; Fig. S2B and S2C). For NanoLuc, pIR-Nluc demonstrated superior performance *in vitro* (Fig. S2D and S2E). *In vivo*, pIR-Nluc outperformed gWiz-Nluc and showed numerically higher values than the commercial Nanoplasmid NTC-9385R backbone (although the difference is not statistically significant) with enhanced expression lasting for at least 4 weeks (Fig. S2F). While additional studies extending beyond 4 weeks will be needed to fully define long-term performance, the pIR platform demonstrated improved persistence relative to the comparator constructs. This improvement is likely attributable to pIR's smaller size, which stems from using a single LoxP site rather than the very large antibiotic resistance elements in the gWIZ vector or the slightly larger RNAOUT selection marker used in the Nanoplasmid system, assuming the regulatory regions remain unchanged. These features translate into significantly better electroporation-based transgene delivery.

Biodistribution studies showed expression largely confined to the injected muscle (Fig. S3 and S4B), with minimal leakage observed only in the lymph node or kidney for the Nluc construct. Blood chemistry, complete blood counts, and cytokine profiling revealed no adverse effects other than a transient increase in MCP-1 at day 7 post-treatment (Fig. S4C–S4L). Histopathology of major organs confirmed the absence of tissue injury (Fig. S5), indicating that pIR delivery is well tolerated.

To assess pIR's utility as a vaccine platform, we encoded mouse LINE-1 ORF1p, a retrotransposon-derived protein of growing interest as a shared cancer antigen. ORF1p is widely expressed across human epithelial cancers yet largely absent from normal adult tissues, making it a promising target for off-the-shelf cancer immunization.^2^ Pir-mOrf1p, engineered with an N-terminal signal peptide^3^ to improve antigen accessibility, exhibited ∼91% supercoiling and a major size reduction (∼3.3 kb) relative to its gWIZ-based counterpart (∼6.2 kb) (Fig. 1E; Fig. S6A). It produced higher antigen levels *in vitro* (Fig. S6A) and, in murine CT26 and MC38 tumor models, induced dendritic cell activation (Fig. S6C and S6D) and significant suppression of tumor growth (Fig. 1F–H; Fig. S6E–S6G) in prime–boost–challenge regimens. These results highlight both ORF1p′s immunogenicity and pIR's suitability for DNA-based cancer vaccines.

To demonstrate the applicability of pIR for therapeutic proteins, we aimed to express GLP1, a small, secreted hormone with strong anti-obesity activity, capable of producing broad systemic effects even at low, steady levels of expression, which makes it an ideal target for pIR-based delivery. However, the native peptide's extremely short half-life poses a major challenge. Current peptide drugs address this by extending half-life through albumin binding or Fc fusion.^4^ Among tested designs that fused GLP1 to either Fc or human albumin, the Fc fusion produced strong GLP-1 secretion *in vitro* (Fig. S7). We then produced pIR-GLP1-Fc (Fig. 1I); and following intramuscular electroporation of pIR-GLP1-Fc at 2 mg/kg, mice maintained circulating GLP-1 concentrations of approximately 500 pM during the first week and ∼200 pM by day 14, with an average of ∼350 pM across two weeks (Fig. 1J). For benchmark comparison, FDA-approved GLP1 peptide drug dulaglutide administered at 0.6 mg/kg yielded a weekly average of ∼200 pM (Fig. 1K).

For the peptide injection, a pronounced early drop in the concentration–time curve indicated rapid systemic clearance and a short circulating half-life, consistent with the drug's reported half-life of approximately four days. In contrast, pIR-mediated GLP1-Fc expression delivered a smoother and more sustained profile, exceeding the functional threshold needed for appetite and glucose control. Prior DNA-encoded GLP-1 studies achieved only 30–60 pM in mouse serum,^5^ underscoring the performance advantage conferred by pIR's size and design. Given that clinical studies have evaluated DNA doses up to 16 mg per injection, the sustained expression profile observed with pIR-GLP1-Fc indicates the potential to explore extended dosing intervals in future studies.

The collective data reinforce the functional importance of vector size for electroporation efficiency. Plasmids above ∼3.5 kb show reduced uptake, slower cytoplasmic diffusion, greater susceptibility to nucleases, and poorer nuclear entry in non-dividing cells such as myofibers. pIR constructs—including pIR-Fluc (3.8 kb), pIR-Nluc (2.8 kb), pIR-mOrf1p (3.3 kb), and pIR-GLP1-Fc (3.0 kb)—all fall within the optimal electroporation window while eliminating antibiotic sequences. The R6K γ origin further reduces the risk of horizontal transfer since replication requires the host-encoded pir gene. Together, these design principles yield a platform that is small, safe, and high-performing, without commercial restrictions that hinder iterative engineering.

Overall, pIR offers compelling advantages for non-viral gene delivery: minimal backbone size, antibiotic-free production, high supercoiling, strong *in vitro* and *in vivo* expression, muscle-restricted biodistribution, and favorable safety. Its performance in cancer vaccine and hormone-replacement applications demonstrates broad therapeutic potential. The system's accessibility and low cost position pIR as a practical tool for academic labs and an attractive vector for translational development. As interest grows in personalized cancer vaccination, long-acting protein therapies, and DNA-based biologics, platforms combining regulatory readiness with experimental flexibility will be essential. pIR provides such a foundation, enabling efficient and scalable DNA delivery across diverse therapeutic modalities.

## CRediT authorship contribution statement

**Dan Zhao:** Writing – review & editing, Writing – original draft, Visualization, Validation, Supervision, Project administration, Methodology, Investigation, Formal analysis, Data curation, Conceptualization. **Zakary D. Carden:** Visualization, Methodology, Investigation. **Yifei Wang:** Methodology, Investigation. **Cole S. Hudson:** Methodology, Investigation. **Perry A. Maza:** Methodology, Investigation. **Dana Cyril-Ramirez:** Project administration. **Yong Li:** Writing – review & editing, Project administration, Funding acquisition, Conceptualization.

## Ethics declaration

All animal procedures were approved by the Institutional Animal Care and Use Committee of Baylor College of Medicine.

## Data availability

All summary data are included within the manuscript. There are no large datasets. Raw data can be provided upon request.

## Funding

This work was supported by grants from the NIH (USA) (No. R01CA278089, U54CA272691 to Y.L.). This study used the Core Facilities and Departmental Shared Equipment resources that are supported by the Dan L Duncan Comprehensive Cancer Center, NIH (USA) (No. P30CA125123), and Cancer Prevention & Research Institute of Texas (CPRIT) Core Facility Awards (USA) (No. RP170005, RP210227). Y.L. is a CPRIT Scholar in Cancer Research (No. RR190043).

## Conflict of interests

Yong Li is an Associate Editor of *Genes & Diseases*. To minimize bias, Yong Li was excluded from all editorial decision-making related to the acceptance of this article for publication. The remaining authors declare no conflict of interests.
