# Supplementary material for: An antibiotic-free plasmid vector platform for non-viral DNA delivery
Source: Genes Dis. 2026 May 23;14(2):102249. doi: 10.1016/j.gendis.2026.102249 (PMC13631514; doi:10.1016/j.gendis.2026.102249)
Supplement: Supplementary file 1 [file mmc1.docx]

**Supplemental Data**

**An antibiotic free plasmid production platform for non-viral DNA delivery**

**This file includes:**

Supplementary Methods and Materials

Supplementary References

Supplementary Tables S1

Supplementary Figs. S1 to S7

**SUPPLEMENTARY MATERIALS AND METHODS**

**Establishment of the pIR producer strain**

The pir-116 strain, which allows high copy propagation of up to 250 copies of R6K origin plasmid per cell, was engineered by integrating arabinose inducible Cre recombinase into the LacZ site so that, upon induction, the parental plasmid is recombined into a backbone reduced minimal circular DNA.

Step 1: Upstream homology arm of LacZ gene (A) was amplified with Lacz-F1/Lacz-R1 primers. Downstream homology arm of lacZ gene (B) was amplified with Lacz-F2/Lacz-R2 primers. Fragments A and B were purified by PCR purification kit (Qiagen, #28104). Using pBADZ-HisCRE plasmid (Addgene, #111187) as template, araC-BAC-CRE was amplified with 0388-F2/0388-R2 primers. PrimeSTAR Max Premix (2X) is used for PCR, with primer concentration at 200 nM and PCR run at 98 ℃ 2min, (98 ℃ 10s, 60 ℃ 10s, 72 ℃ 1min) x 30, 72 ℃ 5 min. Agarose gel electrophoresis please see Fig S1A.

Step 2: In-frame editing fragments consisting of three flanking regions were made by overlap extension PCR. The overlapping fragment was purified by PCR purification kit. PrimeSTAR Max Premix is used for PCR, with primer concentration at 200 nM and PCR run at 98 ℃ 2min, (98 ℃ 10s, 60 ℃ 10s, 72 ℃ 1 min) x 30, 72 ℃ 5 min. Agarose gel electrophoresis please see Fig S1B. Then overlapping fragment was TA cloned into pUC-T vector.

Step 3: pir-116 strain was transformed with pCas9 (Addgene, #42876) and competent pir-116 (pCas9) cells were prepared. Then sgRNA-LacZ and overlapping fragment DNA was electroporated into the pir-116 (pCas9) cells. Positive clones were picked and plated on LB agar plates and the mutated region was amplified with 0388-F2/ lacz-TR11 primers, with an expected size of 3100bp. Agarose gel electrophoresis data please see Fig. S1C. PCR products were sequenced for confirmation. Colony #1, which showed correct PCR sequencing data was further confirmed with whole genome sequencing to confirm the insertion of the arabinose inducible Cre in the LacZ region (Fig. S1D).

Step 4: Western blot confirmation of Cre recombinase induction upon arabinose treatment. 2mM L-arabinose was added to the LB broth for 5 hours at 37 ℃. His-Cre expression was checked with western blot on pre- and post-treatment samples using an anti-His tag antibody (Fig. S1E).

Sequences for above used primers are listed in supplementary table 1.

**Bacterial Growth and DNA Purification Procedures**

Competent pIR producer cells were prepared using the Mix & Go! E. coli transformation kit (Zymo Research, T3001). The pIR parental plasmids were introduced into these cells, and individual transformants were isolated and verified by whole‑plasmid sequencing. Verified clones were cultured overnight in Zeocin (InvivoGen, ant-zn-1)‑supplemented terrific broth (Thermo Fisher, 22711022), after which cell pellets were collected, resuspended in Zeocin‑free terrific broth, and induced with 2 mM L‑arabinose (Sigma, 101492) at 32 °C for 3–4 hours followed by incubation at 37 °C for an additional 1–2 hours to generate the minimal backbone‑free plasmid. Arabinose‑induced cultures were streaked onto plain LB agar to obtain single colonies and miniprep DNA from these colonies was subjected to whole‑plasmid sequencing prior to midiprep or maxiprep production using the endotoxin free ZymoPURE^TM^ kit (Zymo Research, D4201). Whole‑plasmid sequencing using Nanopore technology was performed by QuintaraBio. Purified DNA treated with or without a fast digest restriction enzyme at 37 °C was run on 1% agarose gel. ImageJ (*1*) was used to analyze the supercoil percentage of the final products. For confirmation of cleavage, primers were designed with Primer-BLAST for both the BleoR region or the CMV-intronA region (sequences see Table S1). Realtime PCR were performed using the QuantStudio 7 Pro system and the percentages for uncleaved products were calculated as 100*power (2, Ct^CMVintronA^-Ct^BleoR^).

**DNA constructs**

pBADZ-HisCRE plasmid was a gift from Dr. Tim Richmond (Addgene, #111187) (*2*). pCas9 was a gift from Dr. Luciano Marraffini (Addgene, # 42876) (*3*). All gWIZ-based constructs used in this study were synthesized and provided by Twist Bioscience (South San Francisco, CA, USA). NTC-Nluc is synthesized and provided by Aldevron. All plasmids were confirmed by whole plasmid sequencing.

**Cell lines and DNA Transfection**

293T (CRL-3216), C2C12 (CRL-1772) and CT26 (CRL-2638) were purchased from American Type Culture Collection (ATCC). MC38 (SCC172) was purchased from Millipore Sigma. They were cultured in DMEM with 10% fetal bovine serum (FBS) and penicillin/streptomycin except CT26 (using RPMI1640 plus 10% FBS). All cell lines were authenticated and were regularly tested for mycoplasma contamination. DNA transfection was done using the lipofectamine 3000 transfection reagents (Thermo Fisher, L3000015) following manufacturer’s instructions.

**Experimental Animals**

C57BL/6 and BALB/c mice were obtained from either the Jackson Laboratory or the Center for Comparative Medicine at Baylor College of Medicine. All animal procedures were approved by the Institutional Animal Care and Use Committee of Baylor College of Medicine. Mice were maintained in a temperature‑controlled environment (70 °F ± 2 °F) on a 12‑hour light–dark cycle, provided with 100% fresh HEPA‑filtered air, and given standard chow and water ad libitum.

For DNA electroporation studies, 25–50 µg (approximately 1–2 mg/kg) of DNA was administered into the quadriceps muscle, followed by electroporation using the Ichor TriGrid TDS‑IM array (*4, 5*), which consists of four electrodes arranged in two adjoining equilateral triangles. Blood samples were collected via submandibular puncture into microtainer serum tubes. When indicated, organs and tissues were harvested and processed for protein extraction for NanoGlo assays (Promega, N1120) and BCA assays (Thermo Fisher, A55860) or fixed in formalin and paraffin‑embedded for histological analysis.

For tumor experiments, 7‑ to 9‑week‑old mice received a prime–boost DNA electroporation regimen prior to subcutaneous inoculation with 1 × 10⁶ MC38 or CT26 cells, followed by two additional post‑challenge electroporation. Tumor length (L) and width (W) were measured using calipers, and tumor volume was calculated as ½ × L × W². Tumor wet weights were recorded at the experimental endpoint.

For GLP‑1 studies, mice received either intraperitoneal injections of GLP‑1 peptide at 0.6 mg/kg or intramuscular electroporation with pIR-GLP1-Fc followed by serum collections at the indicated time points. Serum GLP-1 quantification was done using ELISA (Sigma, EZGLP1T-36K).

For all above experiments, dose translation from mice to human was done as instructed from the following paper (*6*).

**Flow Cytometry**

Splenocytes were isolated by mechanical dissociation of freshly harvested spleens through a 70‑µm cell strainer, followed by red blood cell lysis using ACK buffer (Thermo Fisher, A1049201). Single‑cell suspensions were washed and resuspended in FACS buffer and incubated with Fc receptor blocking TruStain FcX (Biolegend, 101320) to prevent nonspecific binding. Cells were then stained with fluorophore‑conjugated antibodies against CD11c (Biolegend, 117336) and DC activation marker CD80 (Biolegend, 104708). After washing, samples were acquired on a flow cytometer, and data were analyzed using FlowJo software.

**Reporter Assays and Bioluminescence Imaging**

For constructs expressing Fluc, bioluminescence imaging was performed using the IVIS Lumina X5 system. For Nluc, NanoGlo assays were carried out following the manufacturer’s instructions (Promega, N1120). For *in vitro* experiments, cells were seeded in culture plates and allowed to adhere overnight. At the indicated time points after transfection, cells were either imaged using the IVIS system (Fluc) or culture supernatants were collected and mixed 1:1 (v/v) with NanoGlo buffer in white, opaque‑walled plates for luminescence measurement (for Nluc) using the CLARIOstar Plus microplate reader (BMG LABTECH, Ortenberg, Germany). For *in vivo* Nluc measurements, serum samples collected from mice were diluted 1:10 in PBS and subsequently mixed 1:1 with NanoGlo buffer prior to plate reading.

**Immunoblots**

Cell lysate preparation and Western blotting were performed as previously described (*7, 8*). Briefly, cells or tissues were lysed using RIPA buffer (Sigma, R0278) supplemented with protease and phosphatase inhibitors (Thermo Fisher, 78441). Protein concentrations were determined using a Pierce Rapid Gold BCA protein assay kit (Thermo Fisher, A55860) according to the manufacturer’s protocol. Cell lysates containing equal amounts of proteins were subjected to 10% SDS-PAGE, transferred to a nitrocellulose membrane, incubated with antibodies, and visualized using Immobilon ECL (Sigma, WBULS0500). The following antibodies were used in the study: His-tag (Proteintech, 66005-1-Ig), GAPDH (CST, #3683), ORF1P (Abcam, ab216324), HRP anti-mouse IgG (CST, #7076), HRP anti-rabbit IgG (CST, #7074).

**Safety Assessments**

For evaluation of pIR safety, day 1 and day 7 post intramuscular electroporation, blood collection was done to measure the white blood cell counts and serum samples were collected to measure organ injury (*9*) with ALT (Raybiotech, MA-ALT-1), AST (Novus Biologicals, NBP3-24470) and creatine (Raybiotech, MA-CTNSP-2) kits or ELISA kits for factors (*10*) below: IL‑6 (Proteintech, KE10007) , TNF‑α (Proteintech, KE10002), IFN‑γ (Proteintech, KE10094), IL‑1β (Proteintech, KE10003), MCP‑1 (Proteintech, KE10123), and GM‑CSF (Biolegend, 432204) following manufacturer’s instructions. Major organs were also collected for histological evaluation. For all ELISAs, analyses were done using the 4-parameter logistic (4PL) method using GainData (arigo Biolaboratories, <https://www.arigobio.com/elisa-analysis>).

**Statistical analysis**

All statistical analysis was performed using GraphPad Prism 10 (GraphPad Software, San Diego, CA). A two-tailed Student’s t test was used to determine statistical differences between two groups, and one-way analysis of variance (ANOVA) was used to compare three or more groups. For repeated measurements and comparison between three groups, two-way ANOVA was used. A *p* value < 0.05 was considered statistically significant.

**SUPPLEMENTARY REFERENCES**

1.	J. Schindelin *et al.*, Fiji: an open-source platform for biological-image analysis. *Nat Methods* **9**, 676-682 (2012).

2.	I. Berger, D. J. Fitzgerald, T. J. Richmond, Baculovirus expression system for heterologous multiprotein complexes. *Nat Biotechnol* **22**, 1583-1587 (2004).

3.	W. Jiang, D. Bikard, D. Cox, F. Zhang, L. A. Marraffini, RNA-guided editing of bacterial genomes using CRISPR-Cas systems. *Nat Biotechnol* **31**, 233-239 (2013).

4.	P. Neeli *et al.*, DNA vaccines against GPRC5D synergize with PD-1 blockade to treat multiple myeloma. *NPJ Vaccines* **9**, 180 (2024).

5.	D. Chai *et al.*, Lipid nanoparticles deliver DNA-encoded biologics and induce potent protective immunity. *Mol Cancer* **24**, 12 (2025).

6.	S. Reagan-Shaw, M. Nihal, N. Ahmad, Dose translation from animal to human studies revisited. *FASEB J* **22**, 659-661 (2008).

7.	D. Zhao *et al.*, Identification of TUBB3 as an immunotherapy target in lung cancer by genome wide in vivo CRISPR screening. *Neoplasia* **60**, 101100 (2025).

8.	D. Zhao *et al.*, Exosomal miR-1304-3p promotes breast cancer progression in African Americans by activating cancer-associated adipocytes. *Nat Commun* **13**, 7734 (2022).

9.	D. Larrey *et al.*, Drug-induced liver injury related to gene therapy: A new challenge to be managed. *Liver Int* **44**, 3121-3137 (2024).

10.	J. M. Tarrant, Blood cytokines as biomarkers of in vivo toxicity in preclinical safety assessment: considerations for their use. *Toxicol Sci* **117**, 4-16 (2010).

**Supplementary Table S1**. **Primers used in this study.**

| Primer ID | Sequence (5’-3’) |
| --- | --- |
| 0388-F2 | TCACACAGGAAACAGCTTGCTACTCCGTCAAGCCGTCAATTGTCTGA |
| 0388-R2 | CGGTTATTACAAAAAGGCCATCCGTCAGGATGGCCTTCTGCT |
| LACZ-F1 | CTGATCATTAACTATCCGCTGGATGACCAGGATGCC |
| LACZ-R1 | AGCTGTTTCCTGTGTGAAATTGTTATCCGCTCAC |
| LACZ-F2 | GCCATCCTGACGGATGGCCTTTTTGTAATAACCGGGCAGGCCATGTCTGC |
| LACZ-R2 | GATCAGTGGCGCAAAGAACATAATCGAGGCGTTAAG |
| LACZ-TF11 | GTCGATGGTAGAACGAAGCGGCGTCGAAG |
| LACZ-TR11 | TTGTCAGAAAGCAGACCAAACAGCGGTTGG |
| BleoR-F | CGACGTGACCCTGTTCATCA |
| BleoR-R | GACACGACCTCCGACCACTC |
| IntronA-F | TATACAACAACGCCGTCCCC |
| IntronA-R | TGTGCTGTGCCTAAGTCTGG |

**Fig. S1. Engineering of the bacteria strain.** (A) Upstream homology arm (A fragment), downstream homology arm (B fragment) and the araC-BAD-Cre fragment were PCR amplified using the PrimeSTAR Max Premix. PCR products were purified and run on agarose gel. (B) Overlap extension PCR was done using the three PCR fragments purified from A and the overlapping fragment was again purified and run on agarose gel. (C) After pCas9 was transformed into the E. coli pir-116 strain and competent cells prepared, sgRNA-lacZ and the overlapping fragment was electroporated into the E. coli pir-116 (pCas9) competent cells to induce integration of the araC-BAD-Cre fragment in the lacZ locus. Single colonies were picked and amplified with 0388-F2/ lacZ-TR11 primers, PCR products were run on agarose gel. (D) Genomic DNA was extracted and whole genome sequencing was performed for colony #1 in C, confirming insertion of the araC-BAD-Cre fragment in the lacZ site (inset). (E) Colony #1 was induced with or without 2 mM L-arabinose for 5 hours. Pre- and post-treatment samples were run on SDS-PAGE and western blotting was done using an anti-His tag antibody.

**Fig. S2. High performances for pIR-encoded luciferase reporters in vitro and in vivo.** (A) Quantifications for 293T (left) or C2C12 (right) from Fig. 1C were shown. (B) IVIS images of mice on days 3 or 14 post electroporation with gWIZ-Fluc or pIR-Fluc. (C) Quantifications for Fig. 1D and S2B were shown. (D) Enzymatic digestion (left) of pIR-Nluc and the maps of pIR-Nluc (middle) vs. NTC-Nluc (right). (E) Nano-Glo luciferase assays of luminescence from 293T cells (E, left) or C2C12 cells (E, right) transfected with pIR-Nluc, gWIZ-Nluc, or NTC-Nluc. (F) Nano-Glo luciferase assays of luminescence using sera collected from mice post electroporation with gWIZ-Nluc, pIR-Nluc, or NTC-Nluc. Unpaired t-tests were used for A and C, One-way ANOVA for E, and two-way ANOVA for F. *p* values were as labeled.

**Figure S3. Ex vivo IVIS imaging post-electroporation with pIR-Fluc.** pIR-Fluc was used for intramuscular electroporation and *ex vivo* bioluminescence imaging were done using the IVIS Lumina X5 system on major organs and tissues.

**Fig. S4. pIR-encoded Nluc expression in vivo is restricted to the injection site.** (A) Scheme of experimental procedures. Tissues or blood samples were collected on day 1 or day 7 post delivery. NanoGlo luciferase assay, ELISA and H&E staining were performed. (B) Nluc activities per µg of proteins from mouse tissues with skeletal muscle as the reference (value = 100). (C) White blood cell counts from animals. (D-F) Colorimetric assays for alanine aminotransferase (ALT; D), aspartate aminotransferase (AST, E), and creatine (F) to assess liver function and kidney health. (G-L) ELISA for serum cytokines, including TNF-α (G), IFN-γ (H), IL-beta (I), IL6 (J), MCP1 (K), and GM-CSF (L). GM-CSF levels were undetectable. One-way ANOVA is used for all panels with *p* values labeled.

**Fig. S5. Histological analysis confirms safety of the pIR platform.** HE stained histology slides display no sign of inflammation or damage in tissues collected from the control (left), 1 day post electroporation (middle), and 7 days post electroporation (right).

**Fig. S6. pIR-ORF1P elicits better immune response than gWIZ-mOrf1p.** (A) Map for gWIZ-mOrf1p (left) Western blot (right) analysis reveals superior expression of mouse ORF1P protein via pIR compared to gWIZ. GAPDH was used as internal control. (B) Scheme of experimental procedures. C57bl/6 mice were injected weekly using electroporation with DNA expressing mouse ORF1P in gWIZ (middle) and pIR (bottom) platforms as well as a mock electroporation control (top). Mice received a prime–boost DNA electroporation regimen prior to tumor inoculation with 10^6^ tumor cells, followed by two additional post‑challenge electroporation. (C) Gating strategy of splenocyte analysis via flow cytometry for dendritic activation. (D) Dendritic cell activation shown as percentage of CD80^+^CD11C^+^ splenocytes. Tumor size measurements (E), tumor image (F) and tumor wet weights (G) were shown for mice inoculated with 1 million MC38 cells in the right flank and treated with mock electroporation, or electroporation with either gWIZ-mOrf1p or pIR-mOrf1p. One-way ANOVA used for D and G, and two-way ANOVA for E. *p* values were as labeled.

**Fig. S7. GLP1-Fc fusion renders better GLP1 expression compared to GLP1-Albumin fusion.** (A) Illustrative maps for GLP1 fusion protein expression cassette using gWIZ. The GLP1 peptide was fused to the human serum albumin (top) or to the human IgG4 Fc (bottom) downstream of a secretion peptide (SP). linker: (G4S)_3_. (B) 293T (left) or C2C12 (right) cells were transfected with the GLP1 fusion protein constructs and cell culture supernatants were collected 48h post transfection. ELISA against GLP1 was performed and data analyzed via the 4-parameter logistic method. Two-tailed unpaired t-tests were used for B. *p* values were as labeled.
